# A mechanical perspective on suction feeding in fishes

**DOI:** 10.1242/jeb.250567

**Published:** 2025-09-30

**Authors:** Ariel L. Camp, Sam Van Wassenbergh

**Affiliations:** ^1^ Department of Musculoskeletal and Ageing Sciences, Institute of Life Course and Medical Sciences, University of Liverpool, Liverpool L7 8TX, UK; ^2^ Laboratory of Functional Morphology, Department of Biology, University of Antwerp, Universiteitsplein 1, 2610 Antwerpen, Belgium

**Keywords:** Power, Muscle, Fluid dynamics, Skeletal linkage

## Abstract

Suction feeding in fish has long fascinated experimental biologists because of its complex motions, intricate anatomy and vast distribution across thousands of species and nearly every aquatic habitat. Suction feeding poses three main mechanical challenges for fish. First, how do shortening muscles create three-dimensional (3D) expansion of the mouth cavity to suck in water? Second, how do muscles produce the substantial power required for fast and forceful expansion to accelerate food and water into the mouth? Third, how is water moved through the mouth so that food can be deposited in the oesophagus? Over the last 15–20 years, new methods for measuring and modelling bone, muscle and fluid motions have advanced our understanding of how fish meet these three mechanical challenges of suction feeding. In this Review, we examine these advances, primarily through the lens of mechanical power, and highlight understudied areas with exciting new questions. We discuss how skeletal levers and linkages transform and transmit muscle force into 3D mouth cavity expansion. We explain how the power for rapid and forceful expansion is generated primarily by large regions of the body muscles – although, for all feeding muscles, power output depends on how much and how fast the muscles shorten. Finally, we outline the key characteristics of flows outside and inside the mouth, and their implications for food capture and transport. Future research into the interactions of skeletal motion, muscle function and water flows will reveal new insights into suction-feeding morphology, evolution and ecology.

## Teaser

**Summary:** We review the mechanics of how fish feed by creating powerful suction through the interplay between muscle function, skeletal force transmissions and fluid flow patterns.

## Introduction

Suction feeding in fish is a distinctive musculoskeletal behaviour that has captivated experimental biologists for nearly 100 years (Gregory, 1933; Roberts-Hugghis et al., 2025). During suction feeding, fish expand the mobile cranial skeleton surrounding the mouth cavity rapidly (usually 4–40 ms; Gibb and Ferry-Graham, 2005) and in all three dimensions (Alexander, 1967a; Lauder, 1980; Muller et al., 1982; van Leeuwen, 1984; Camp et al., 2015; Whitlow et al., 2022). As water is virtually inextensible, the expanding cavity will cause water and food to flow from outside into the open mouth. Typically, the mouth reaches its maximum opening just as food enters (Alexander, 1967a; Holzman et al., 2007; Day et al., 2015). Water continues to flow into the mouth and then exit through the opercular slits while food is retained inside the mouth cavity. After this capture phase, the mouth returns to its initial position (recovery phase), after which smaller motions may reposition the food before it is eventually swallowed (transport phase) (Provini et al., 2022; Sibbing et al., 1986; Weller et al., 2020).

Compared with other vertebrate feeding methods, suction feeding is remarkable for its mechanical complexity, morphological disparity and phylogenetic and ecological versatility. It involves over 30 moving bones and their associated muscles and ligaments (Anker, 1974; Liem, 1970, 1978), whose shape varies dramatically across species (Gregory, 1933; Westneat, 2006; Troyer et al., 2024), and is used by most of the over 30,000 living fish species in nearly every aquatic habitat and feeding niche (Corn et al., 2022; Longo et al., 2016; Wainwright et al., 2015). Suction feeding is of great interest to functional morphologists and is also a useful system for discovering general principles of macroevolution (Evans et al., 2021; Longo et al., 2016; Roberts-Hugghis et al., 2025), vertebrate development (Hu and Albertson, 2014; Hulsey et al., 2005; China et al., 2017) and genotype–phenotype relationships (Albertson et al., 2005). A rich literature covers suction-feeding anatomy, kinematics, muscle activation and hydrodynamics (reviewed in Day et al., 2015; Ferry-Graham and Lauder, 2001; Lauder, 1985; Wainwright et al., 2015). However, integrating these to understand what drives suction-feeding performance remains a challenge (Holzman et al., 2012; Wainwright et al., 2007).

**Fig. 1.**
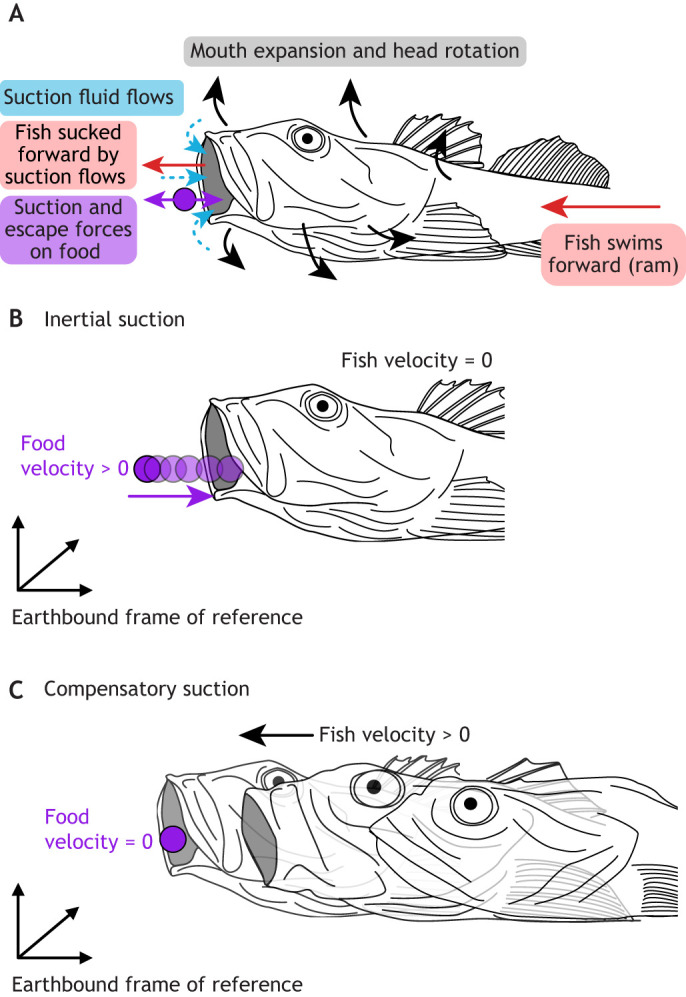
**Dynamics of suction feeding result from the interaction of musculoskeletal, fluid and food forces and movements.** (A) Lateral view of suction feeding. Movements of the fish – forward motion, head rotation and mouth expansion (solid black and red arrows) – generate suction flows (dashed blue arrows) into the mouth cavity. Fluid motion exerts forces on the food (purple arrow). Suction flows accelerate food towards the mouth, while the displacement of water by the fish's forward motion (i.e. the ‘bow wave’) will push food away from the mouth. Suction feeding in fish will always be a combination of two extreme situations (see also Longo et al., 2016; Norton and Brainerd, 1993). (B) In pure ‘inertial suction’, the fish is immobile while water and food are accelerated into the mouth. (C) In ‘compensatory suction’, the fish expands the head (to overcome the bow wave) while moving forward as water and food remain immobile in the earth-bound reference frame.

Many open questions remain on the mechanics of suction feeding: how muscles, the skeleton and fluid forces work together to create suction. These are key for understanding how fish overcome three main challenges of suction feeding (Fig. 1). First, fish must expand the mouth cavity using motors (i.e. skeletal muscles) that can only power contraction. Therefore, a mechanical system is needed to transform muscle shortening into mouth cavity expansion: a complex system of joints and linkages (see Glossary) (Anker, 1974; Tchernavin, 1948; Westneat, 2004; Whitlow et al., 2022). Second, suction requires substantial power: it must be both fast and forceful to accelerate fluid and food into the mouth. Fish using direct muscle contraction require up to 800 W kg^−1^ of instantaneous power during suction expansion (Camp and Brainerd, 2022; Avidan et al., 2023). How do muscles produce the skeletal motions that generate such powerful suction flows? Third, outflow of water must be permitted to enable a sufficiently persisting flow towards the back of the mouth cavity, without the risk of losing food in the exiting water (van Leeuwen and Muller, 1984; Provini et al., 2022). How fish address these three challenges through their intricate transmission systems for generating mouth cavity expansion driven by high muscle power, remains an active area of research.

Analytical and computational models have generated and tested hypotheses answering these questions. These models integrate anatomical and behavioural observations with quantitative tools (e.g. Aerts and Verraes, 1984; Alexander, 1970; Carroll et al., 2004; de Lussanet and Muller, 2007; De Visser and Barel, 1998; Muller et al., 1982; Van Wassenbergh and Aerts, 2008). However, limitations in biological measurements and computational power – leading to assumptions or limited interpretation – mean many of these hypotheses have not been fully tested or developed. Over the last 15–20 years, new methods have emerged to directly measure kinematics, muscle dynamics and fluid dynamics with greater detail, in more species, during live (or more life-like) behaviours (e.g. Avidan and Holzman, 2021; Camp and Brainerd, 2022; Jimenez and Brainerd, 2020; Provini et al., 2022). These innovations in experimental methods and computing power foretell potentially substantial advancements.

In this Review, we discuss the musculoskeletal and fluid dynamics of suction feeding mainly through the lens of mechanical power. Mechanical power is the rate of energy change and can be calculated as the product of force and velocity (e.g. in skeletons or muscles) or the product of the rate of change of volume change and pressure (e.g. in a fluid system). As power is crucial for explosive actions such as suction feeding, it has been a major focus in the discovery of mechanisms and used as a proxy for performance (Avidan et al., 2023; Camp et al., 2015; Longo et al., 2018; Van Wassenbergh et al., 2008), in analogy with studies in terrestrial and aquatic locomotion (Altringham and Johnston, 1990; Askew and Marsh, 2001; Peplowski and Marsh, 1997; Roberts, 2019). Mechanical power is relevant to understanding skeletal motion, muscle behaviour and hydrodynamics – and therefore the mechanical challenges of suction feeding. Now, researchers have more tools than ever to calculate and model power in suction-feeding fish. Our Review (see sections below) is organized around the roles of the skeleton (‘Skeletal levers and linkages of mouth expansion’), the muscles (‘How muscles generate the power for suction feeding’) and the fluid forces (‘The interaction of musculoskeletal systems and fluid forces’) in generating and transmitting suction-feeding power.

Although feeding involves many phases, this Review focuses on the generation of suction flows and their role in food capture by teleost fishes. This capture phase involves fluid flows from mouth expansion that draw food into the mouth cavity. This phase is the best known from a mechanical perspective, allowing the best opportunity for synthesis. Suction is rarely the sole means of prey capture (Fig. 1B,C). Translation (see Glossary) of the whole fish (‘body ram’, Fig. 1C), rotation of the head (pivot feeding) and/or translation and rotation of the anterior jaws (‘jaw ram’) towards the food also contribute to successful feeding (Longo et al., 2016; Norton and Brainerd, 1993). Where these feeding modes overlap mechanically with suction feeding (e.g. head rotation and anterior jaw motion), they are discussed. We examine suction feeding primarily in teleost fish (‘fish’ hereafter), as this group includes most of the anatomical, kinematic, ecological and phylogenetic diversity of aquatic suction feeding (Wainwright et al., 2015). Powerful suction feeding is also employed by cartilaginous fishes (Wilga et al., 2007), amphibians (Dean and Lannoo, 2003; Deban and O'Reilly, 2005) and mammals (Marshall et al., 2008). However, many of these groups use fundamentally different musculoskeletal systems and fluid dynamics. The timing and magnitudes of skeletal motion, muscle power and flow speeds vary substantially with fish size and species, so we focus on describing the patterns of suction mechanics, rather than the numerical values.

Glossary **Abduction**

Moving an anatomical structure laterally, away from the midline of the body. The opposite motion is adduction.

**Adduction**

Moving an anatomical structure medially, towards the midline of the body. The opposite motion is abduction.

**Axial muscle**

Muscles along the trunk and tail, including both epaxial (dorsal) and hypaxial (ventral) regions.

**Branchial basket**

Anatomical structures between the mouth cavity and the oesophagus, including the gill arches, gill rakers and pharyngeal jaws.

**Epaxial muscles**

Dorsal trunk muscles, surrounding the vertebral column and extending from head to tail in fish.

**Force couple**

A mechanical system with a pair of forces with equal magnitudes and opposite directions, resulting in only rotational motion.

**Gearing system**

A mechanism that increases angular velocity at the cost of a reduction in torque (or vice versa).

**Hyoid symphysis**

The joint linking the left and right hyoid bones (ceratohyals).

**Hypaxial muscles**

Ventral trunk muscles, surrounding the vertebral column and extending from head to tail in fish.

**Linkage**

Bones connected by joints or soft tissues so that multiple bones move together

**Neurocranium**

Part of the skull skeleton which surrounds the brain and eyes, forming the ‘roof’ of the mouth cavity.

**Particle image velocimetry (PIV)**

A technique to visualize and measure the velocity of fluid flow by tracing the motion of suspended particles.

**Shear**

Deformation of a material where parallel planes slide past each other resulting in a change in shape, rather than length.

**Strain**

The relative deformation of a material, often calculated as the ratio of the change in length to the original length.

**Streamline**

The path of a (massless) particle moving with a fluid flow.

**Translation**

Straight-line motion or displacement of a structure (as opposed to pivoting or rotation).

## Skeletal levers and linkages of mouth expansion

**Fig. 2.**
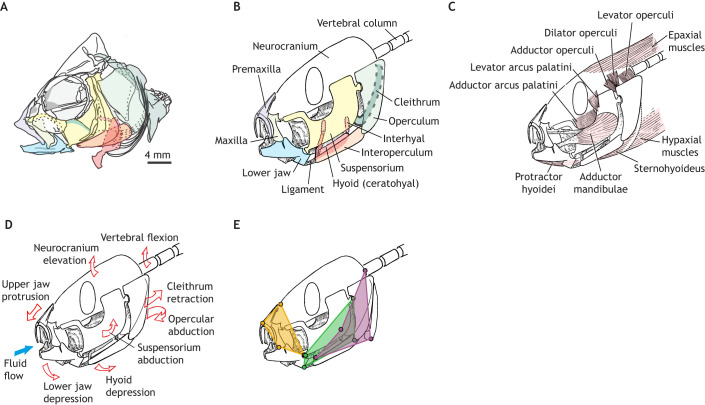
**Musculoskeletal components of suction feeding.** (A) Anatomical drawing of the skull of *Haplochromis elegans* (after Barel et al., 1976), on which the diagrams in B–E are based. Colours correspond to the labelled bones in B. (B) Independently mobile bones that contribute to mouth cavity expansion. (C) Key muscles used in suction feeding. (D) Skeletal motions (red arrows) and fluid flow (blue arrow) that can contribute to mouth expansion. (E) Main skeletal linkages for cranial expansion: opercular linkage (green), anterior jaw linkage (orange) and neurocranium–hyoid linkage (purple); see main text for details. B–E are based on illustrations by Peter Aerts, with permission.

The exceptionally mobile feeding apparatus of fish transmits muscle power to the fluid environment and transforms muscle contraction into mouth cavity expansion. Vertebrate skeletal muscles only generate power by contracting, i.e. actively shortening, so they cannot directly power expansion. Instead, the feeding skeleton acts as a system of levers to transform muscle shortening into mouth cavity expansion (Fig. 2). In fish skulls, most of these levers are connected into linkages so that motions of cranial elements are coupled, and a single muscle can move multiple – and sometimes distant – bones (e.g. Anker, 1978; Muller, 1989; Van Wassenbergh et al., 2005b; Westneat, 1990). Levers and linkages can transform the direction and the ratio of force and velocity of muscle contraction, which may indirectly change muscle power, by altering the lengths and velocities at which the muscle operates (see ‘How muscles generate the power for suction feeding’, below). Linkage kinematics determine the shapes and surfaces that interact with fluid forces (see ‘The interaction of musculoskeletal systems and fluid forces’, below). Thus, the anatomy and motion of these linkages are key to understanding suction-feeding mechanics and the flow of energy through the feeding apparatus and surrounding fluid. In the sections below, we describe the most common skeletal linkages known in fish feeding, with a particular focus on the hyoid apparatus. The relative timing of these motions varies substantially with species and size (see Gibb and Ferry-Graham, 2005; Day et al., 2015; Whitlow et al., 2022).

### Neurocranium and pectoral girdle

Rotation of the neurocranium (see Glossary) and pectoral girdle contributes directly to expanding the mouth cavity and transmitting power from the body muscles to the feeding apparatus (Fig. 2). Dorsal rotation (elevation) of the neurocranium and caudoventral rotation (retraction) of the pectoral girdle (Fig. 2D) increase the dorsoventral height of the mouth cavity (Lauder, 1985; Tchernavin, 1948). Together, these motions can also contribute to every other mouth expansion linkage (Fig. 2). Neurocranium and pectoral girdle rotations, relative to the fish's body, can only be produced by active shortening of the epaxial and hypaxial muscles (see Glossary; Fig. 2C). Generally, epaxial and hypaxial power cannot be transmitted to the rest of the feeding apparatus without rotating the neurocranium and pectoral girdle, respectively.

While these motions are common, they are not universal in suction feeding (Camp and Brainerd, 2022; Lauder, 1985; Westneat, 2006). Species without neurocranial elevation and/or pectoral girdle retraction may generate successful but less powerful strikes (Camp and Brainerd, 2022). However, even as a stable anchor, the neurocranium can play an important role in the forces of suction expansion (Jimenez et al., 2023). The skeletal mechanisms of neurocranial elevation and pectoral girdle retraction will impact the loading forces and length dynamics – and therefore power output – of the body muscles (see also ‘How muscles generate the power for suction feeding’, below). In most fish, neurocranial elevation is probably the result of dorsal rotation (flexion) across the craniovertebral and multiple intervertebral joints (Camp, 2021; Ford, 1937; Jimenez et al., 2018; Tchernavin, 1948; Van Wassenbergh et al., 2011). Recent measurements have shown that large regions of the vertebral column can flex to achieve neurocranial elevation (Camp, 2021). Pectoral girdle retraction can result from rotation of the joints within the girdle and/or between the girdle and the neurocranium (Camp and Brainerd, 2014; Gosline, 1977). Pectoral girdle joint motions and morphology remain poorly understood but intriguing areas for future research (Holcroft and Wiley, 2015).

### Lower jaw depression

**Fig. 3.**
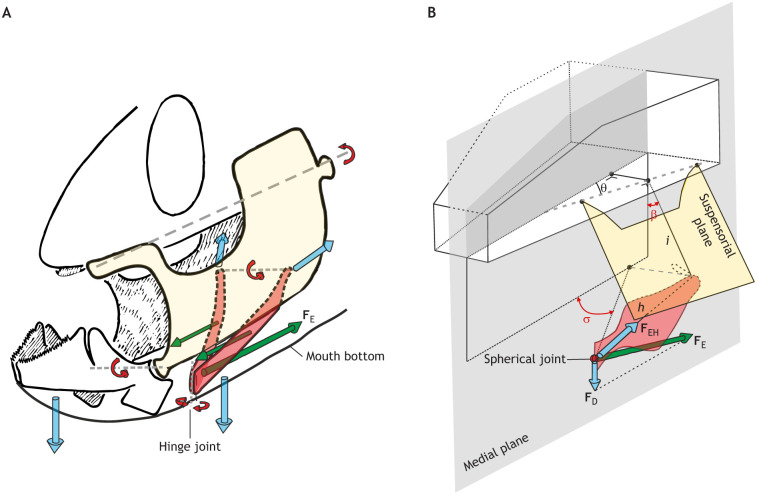
**Force transmission by the hyoid.** (A) Schematic diagram of the model of Aerts (1991) in which the symphysis of the hyoid (red) is treated as a hinge joint, and an input force couple is exerted on each hyoid bar (green arrows; **F**_E_, expansive force through the sternohyoideus; forward-pointing arrows represent forces from the protractor hyoidei and the ligamentous connections between the hyoid and mandible). The blue arrows show head expansions due to hyoid forces: mouth opening, buccal floor depression and suspensorium (yellow) abduction. Based on illustrations by Peter Aerts, with permission. (B) The kinetic model by de Visser and Barel (1998) showed that two rotational states (hyoid depression angle σ, and hyoid–suspensorium abduction/adduction angle β) and three constructional parameters (*i*, *h*, θ) influence the mechanics of the hyoid–suspensorium system. The hyoid symphysis is treated as a point (spherical) joint so that **F**_E_ resolves into a depressing force **F**_D_ and a force along the hyoid bar **F**_EH_. **F**_EH_ is transferred to the suspensorium to cause its rotation. Modified after de Visser and Barel (1998).

Jaw opening is a key step in beginning the process of mouth expansion and allowing water to flow into the mouth cavity. Fish have multiple mechanisms for jaw depression: the opercular linkage (Fig. 2E, green), the hyoid linkages (Fig. 2E, purple; Fig. 3) and shortening the protractor hyoidei muscles (Fig. 2C) (Adriaens et al., 2001; Gosline, 1971; Van Wassenbergh et al., 2005b). In the opercular linkage, rotation between the operculum and suspensorium causes caudal motion of the suboperculum–interoperculum joint, which pulls on the interoperculomandibular ligament, generating caudal motion of the articular process and ventral rotation (depression) of the lower jaw (Fig. 2B–D). Originally, this linkage was hypothesized to be driven by the levator operculi muscle dorsally rotating the operculum (Liem, 1970) and modelled as a planar four-bar linkage (Anker, 1974; Barel, 1977). These models and hypotheses were tested when the 3D skeletal kinematics of the opercular linkage were measured in largemouth bass (*Micropterus salmoides*) (Camp and Brainerd, 2015). They found this linkage was instead powered by the epaxial muscles rotating the neurocranium and operculum relative to the suspensorium. The levator operculi muscle held the operculum in place as the neurocranium and suspensorium rotated dorsally away (Camp and Brainerd, 2015). This demonstrates that the cranial muscles can shape how axial muscle (see Glossary) power is transmitted through the skull and transformed into mouth expansion (Elshoud-Oldenhave, 1979). The opercular linkage allows anterior expansion (lower jaw depression) to be powered by posterior muscles (the epaxials) independently of hyoid movement. This independence allows phase shifts between jaw and hyoid depression (Otten, 1982), so that mouth opening can start and peak before hyoid motion, resulting in an anterior-to-posterior wave of expansion. The independence of lower jaw and hyoid kinematics is evident in recent studies of 3D skeletal kinematics of suction feeding (Whitlow et al., 2022, 2025).

### Jaw protrusion

Many fish rotate and/or translate the upper jaw forward as the mouth opens. This jaw protrusion contributes to successful suction feeding by moving the jaws closer to the prey, i.e. jaw ram (Longo et al., 2016), and by creating a more circular mouth aperture, which increases suction flow speeds and fluid forces on prey (Holzman et al., 2008; Skorczewski et al., 2012). Jaw protrusion cannot be directly generated by muscles alone. Instead, fish use a variety of linkage mechanisms to transform dorsoventral rotation (jaw depression and or neurocranium elevation) into rostral rotation and/or translation of the upper jaw (Alexander, 1966; Eaton, 1935; Gidmark et al., 2012; Gregory, 1933; Liem, 1980; Staab et al., 2012; Westneat and Wainwright, 1989). These linkages rely on both bone motion and the ligaments and/or skin that link the upper and lower jaws (Alexander, 1967b; Westneat, 1990).

### Hyoid kinetics

The hyoid apparatus is crucial during the expansive phase of suction feeding by serving as a key component in transmitting forces that lead to head expansion (e.g. Aerts, 1991; Anker, 1974; De Visser and Barel, 1996, 1998; Muller, 1987; Van Wassenbergh et al., 2013). To do so, the hyoid distributes the forces towards expansion in three directions: (1) mouth-bottom depression, (2) mouth opening and (3) suspensorium abduction (Fig. 3). The hyoid ‘bars’ (i.e. the ceratohyal bones) can be regarded as the left and right components of a pair of converging rods (De Visser and Barel, 1996). To simplify our view on the kinetics of the hyoid, the neurocranium is treated as the fixed element. In that case, a single expansion force (**F**_E_) originates from the retraction of the pectoral girdle, transmitted by the sternohyoideus muscle to the hyoid symphysis (see Glossary; Fig. 3). This force (**F**_E_) is typically oriented to rotate the hyoid counterclockwise (fish facing left), causing the hyoid symphysis to move ventrally and depress the mouth bottom (Fig. 3). The hyoid may also be retracted slightly (i.e. translated posteriorly) in fish with an interhyal or a relatively long ligament at the connection to the suspensorium (Fig. 3; Adriaens and Verraes, 1994). While rotating ventrally and retracting, the hyoid assists in depressing the lower jaw by pulling either the protractor hyoidei muscles or the mandibulo-hyoid ligaments. Finally, the forces on the hyoid in the sagittal plane (**F**_E_, and forces to and from the lower jaw), will cause laterally directed forces on the suspensoria, generating abduction (see Glossary; Fig. 3). Consequently, powerful rotations of the neurocranium and pectoral girdle in the sagittal plane are converted to lateral expansions via forces on and by the hyoid.

The transmission of forces by the hyoid depends on many factors, all of which are critical for generating effective suction. Force-transmission modelling studies on sticklebacks (Anker, 1974) and cichlids (Aerts, 1991; De Visser and Barel, 1998) revealed the importance of a list of geometrical properties of the hyoid–suspensorium apparatus (Fig. 3B). In cichlids, the geometries found in specialist piscivores allow the expansive force (**F**_E_; Fig. 3) to cause early mouth opening without suspensorium abduction. This causes a brief adduction (see Glossary) of the suspensoria, as observed *in vivo* (‘preparatory phase’; Liem, 1978), and later produces strong abduction forces when the mouth is opened sufficiently wide (De Visser and Barel, 1998). Broad-headed fish, such as algae-grazing cichlid species with enlarged jaw adductors, do not show optimal conditions for expansion, indicating a trade-off with biting performance (De Visser and Barel, 1998). The hyoid symphysis joint properties are important as well. A hinge joint can allow a force couple (see Glossary) in the antero-posterior direction, pulling on the hyoid to result in lateral forces on the suspensoria (Aerts, 1991). This effect allows seahorses to widen their snout even with the hyoid rotated over 90 deg (Van Wassenbergh et al., 2013; see ‘Amplifying muscle power’, below).

## How muscles generate the power for suction feeding

Skeletal linkages transform and transmit force and velocity through the feeding apparatus, but it is the muscles of suction feeding that generate force through contraction, producing power as the product of force and contraction velocity. Suction feeding is predicted to be a power-limited behaviour in many fish (Carroll and Wainwright, 2006; Coughlin and Carroll, 2006; de Jong et al., 1987; Van Wassenbergh et al., 2005a). Suction expansion shows instantaneous muscle power outputs averaging 129 W kg^−1^ across species (Avidan et al., 2023), and up to 200–800 W kg^−1^ for expansions in high-performance suction feeders (Camp and Brainerd, 2022). Therefore, understanding how the muscles of mouth expansion generate power and what may limit their power production provides important insights into suction-feeding mechanics. To generate power for suction feeding, muscles must: (1) have anatomical insertions sufficient to generate expansion motions, (2) be electrically active, i.e. generating force, and (3) be shortening, i.e. generating positive velocity. All the muscles of suction feeding (Fig. 2C) meet the first two criteria (Alfaro et al., 2001; Lauder, 1985; Westneat, 2006). However, only recently have muscle lengths been measured in multiple muscles – and throughout the body muscles – together with suction power to determine which muscles actively shorten during peak suction expansion power (reviewed in Camp and Brainerd, 2022). Here, we summarize recent data on muscle length dynamics and their interaction with muscle force–length and force–velocity properties. Together, these provide new insights into how fish power suction expansion, what limits suction power and, ultimately, how suction flows are generated.

**Fig. 4.**
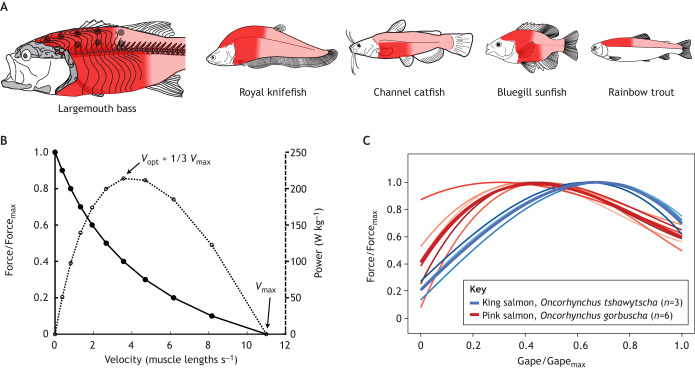
**Muscle shortening and length dynamics impact suction power.** (A) The axial muscles (light pink) actively shorten over large regions (dark red) to generate power during suction feeding. In largemouth bass, muscle shortening (from Camp and Brainerd, 2014) and muscle activation (grey circles; from Thys, 1997) have been measured over large regions of the axial muscles during suction feeding. The same colour scheme shows the extent of muscle shortening during suction feeding in four additional species (from Camp et al., 2018, 2020; Jimenez and Camp, 2023; Li et al., 2022). For rainbow trout, only epaxial muscles were measured. (B) Estimated force–velocity (solid line, filled circles) and power–velocity (dotted line, open circles) relationships for largemouth bass epaxial muscles, based on published values of maximum velocity (*V*_max_), maximum power and optimum velocity for power production (*V*_opt_) of about 1/3 of *V*_max_ (Coughlin and Carroll, 2006) and a maximum muscle force of 159 kN m^−2^ (James et al., 1998). A Hill-type force–velocity curve was fitted to these values, as in Camp et al. (2015). (C) The relationship between gape and jaw-closing (adductor mandibulae) muscle force (both relative to their maxima) in two salmon species reflects the force–length relationship of muscle. The greatest muscle forces are generated at intermediate gapes. Thick lines represent regressions fitted to each species, thin lines represent regressions fitted to each individual (each shade of red or blue represents a different individual). Adapted, with permission, from Kaczmarek and Gidmark (2020).

The body muscles have the potential to generate much more power than the comparatively small cranial and hypobranchial (sternohyoideus) muscles (Aerts et al., 1987; Elshoud-Oldenhave, 1979; Tchernavin, 1948). If all else is equal, a large muscle has a greater maximum theoretical power output than a small one. But this potential power is only realized if large regions of the body muscles actively shorten during suction feeding. As the body muscles extend from head to tail, there is no obvious morphological delineation of which regions shorten during feeding. Muscle shortening has now been measured in subregions throughout the dorsal (epaxial) and ventral (hypaxial) body muscles in five species with X-ray video (i.e. fluoromicrometry; Camp et al., 2016; Camp and Brainerd, 2022; Jimenez and Camp, 2023), showing over 80% of the axial muscle mass shortens during suction expansion (Fig. 4A). This shortening is assumed to be active, as there are no known mechanisms for passive shortening of these muscles. In largemouth bass, epaxial muscle activity has been measured over the cranial half of the musculature during suction feeding (Thys, 1997). Large regions of the body muscles generate over 90% of the power for suction expansion during powerful strikes (Camp and Brainerd, 2022).

The actual magnitude of power produced by the body muscles depends on multiple factors including activation patterns and dynamics, length dynamics and the molecular composition of muscle fibres. Fish can vary activation within and across segments of the body muscles (Thys, 1997) to adjust their suction-feeding performance – and probably power – by increasing the volume of body musculature that is activated (Carroll and Wainwright, 2006; Jimenez and Brainerd, 2020, 2021). The timing of muscle activation (relative to the timing of muscle force production and loading of external forces) will also impact muscle power output, although this has only been investigated in the epaxial muscles of largemouth bass (Coughlin and Carroll, 2006). While the body muscle regions contributing to suction feeding are composed of fast, white fibres, these fibres can have different proteins that impact their force and velocity production (Coughlin, 2002; Coughlin and Akhtar, 2015; Rome et al., 1993). However, this molecular variation has not been studied in the context of the feeding function of these muscles. We expect that investigating how these physiological properties impact suction-feeding performance will be a productive area of future study, with the possibility to integrate the substantial literature on the physiology of fish body muscles during swimming. Below, we focus on the role of muscle length dynamics, as the most data exist for this factor.

### Muscle length and speed shape power output

The power output of suction expansion muscle, like that of all skeletal muscles, depends on the magnitude and rate of muscle shortening. Muscles have a trade-off between force and velocity, such that maximum power is only produced when the muscle shortens at intermediate velocities (Fig. 4B). The velocity for maximum power output (*V*_max_) has been measured in the feeding muscles of a few species (Carroll et al., 2009; Van Wassenbergh et al., 2007b), with some evidence that cranial muscles (such as the levator operculi and sternohyoideus) may operate near *V*_max_ during suction feeding (Camp et al., 2018; Lomax et al., 2020). Shortening at a uniform velocity near *V*_max_ is more challenging for the body muscles. During cranial elevation for suction feeding, the fish's body bends like a beam with a gradient of strain (see Glossary) as measured along the craniocaudal line of action (Jimenez and Camp, 2023; Jimenez et al., 2021), similar to lateral bending during swimming (Muller and van Leeuwen, 2006). If the axial muscle fibres ran along this line of action, parallel to the vertebral column, shortening velocity would vary throughout the muscles, with only a small portion of fibres shortening at *V*_max_. Fish axial muscle fibres are not arranged parallel to the vertebral column, but have a complex 3D, pseudo-helical orientation (Alexander, 1969; Gemballa and Vogel, 2002; van Leeuwen, 1995; van Meer et al., 2025). The fibre orientation and shear (see Glossary) deformation of body muscles are hypothesized to homogenize medio-lateral strain during lateral bending (Alexander, 1969; van Leeuwen et al., 2008). However, this has not been directly measured, and it is unknown whether the same mechanism can homogenize dorsoventral strain gradients (Jimenez et al., 2021). The complex architecture of fish body muscles has made it nearly impossible to directly measure muscle fibre shortening during live behaviours (e.g. Goldbogen et al., 2005). A major challenge is to advance our current measurements of body muscle strain during feeding – which only captures shortening along the line of action craniocaudally – to determine epaxial and hypaxial fibre shortening.

Muscle function also depends on the length at which it contracts: muscles only produce their maximum force over a small range of intermediate lengths (e.g. Peplowski and Marsh, 1997). By operating at the optimal length for force production (*L*_o_), muscles can generate more force for a given shortening distance, and therefore potentially more power. *L*_o_ has only been measured in a few fish feeding muscles in the context of biting (Fig. 4C), with the interesting result that muscles may not always be operating at or near *L*_o_ (Gidmark et al., 2013; Kaczmarek and Gidmark, 2020). In suction feeding, this force–length relationship may be particularly relevant for the comparatively long, parallel-fibred protractor hyoidei and sternohyoideus muscles (Fig. 2C). These muscles attach directly to the hyoid apparatus and may undergo substantial length changes as it moves (Camp et al., 2018; Lomax et al., 2020; Van Wassenbergh et al., 2005b, 2007a) and play an important role in contributing force that is then transmitted throughout the skull via the hyoid linkage (Fig. 3). Through these force–length and force–velocity relationships, skeletal linkages may impact muscle power production. If the leverage of these linkages is tuned to the properties of the muscles actuating them, they may act as a gearing system (see Glossary), allowing muscle to operate at the optimal lengths and velocities to maximize power output (e.g. Richards and Clemente, 2013). We encourage more studies linking muscle function and skeletal motion during suction feeding to test this exciting hypothesis.

### Amplifying muscle power

Given the importance of power from the suction feeder in swiftly capturing its prey by suction, it is unsurprising that some suction-feeding fish targeting evasive prey (such as small crustaceans) have evolved methods to generate higher instantaneous power outputs (about 2000–8000 W kg^−1^; Van Wassenbergh et al., 2008; Longo et al., 2018; Avidan et al., 2023) than is possible through muscle contraction alone (1121 W kg^−1^; Askew and Marsh, 2001). This is the case for the clade of syngnathiform fishes. This group of long-snouted species includes Syngnathidae (seahorses, pipefish, seadragons) and their closest relatives, such as cornetfish, trumpetfish and snipefish (Longo et al., 2017). Syngnathiforms depend on an elaborate dorsal pivoting of their head and relatively long snout to bring their mouths close to their prey in only a few milliseconds before suction engulfs them. This strategy is referred to as pivot feeding (de Lussanet and Muller, 2007).

**Fig. 5.**
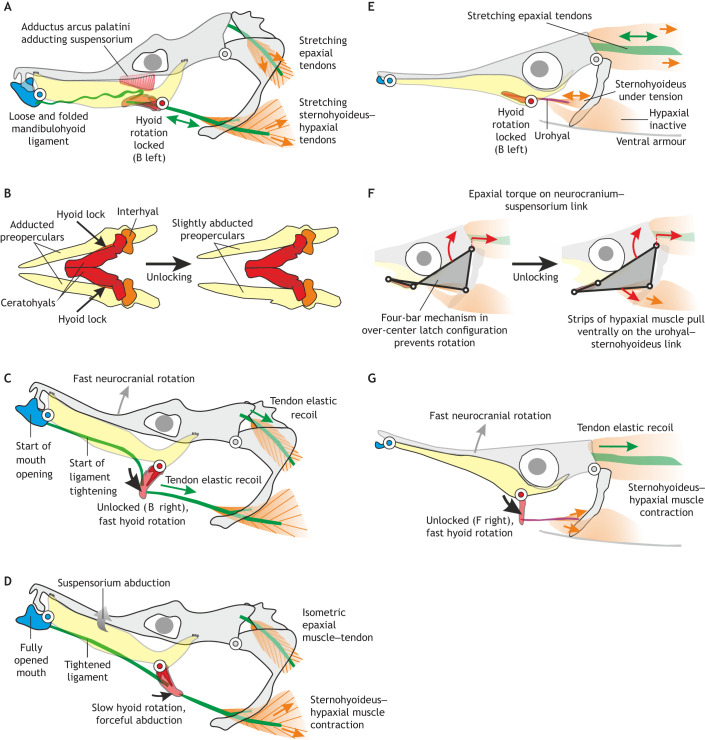
**Power-amplified suction-feeding in a seahorse (left) and snipefish (right).** Circles represent the three joints allowing rotation in the sagittal plane (grey, occipital joint; red, hyoid joint; blue, quadratomandibular joint). (A,E) During the preparatory phase, the epaxial muscles (orange) contract (Muller, 1987; Van Wassenbergh et al., 2008). Because the hyoid is prevented from rotating ventrally at this stage, the epaxial tendons (green) are stretched without causing any skeletal motion. In seahorses, the sternohyoideus–hypaxial muscles (orange) also contract during this phase (Van Wassenbergh et al., 2014) and stretch their tendon. The mechanism for locking the hyoid is hypothesized to vary. (B) In seahorses (ventral view), a small ventromedial part of the adducted preopercular bones lies ventral to the lateral sides of the posterior ceratohyals (left) (de Lussanet and Muller, 2007); relaxation of the adductor arcus palatini muscles would then allow the ceratohyals to pass ventrally in between the left and right preopercular bones (right). (F) In snipefish, the hyoid is hypothesized to be locked by a four-bar mechanism including an over-centre latch of the neurocranium–hyoid linkage: as long as the urohyal–sternohyoideus link is dorsal to the hyoid, epaxial torque will further push the hyoid in its maximally elevated position (left). The hypaxial muscles may then act as a trigger by pulling the urohyal–sternohyoideus link below the hyoid, unlocking the hyoid to rotate ventrally (right). (C,G). This simultaneously causes fast rotations of the neurocranium and the hyoid (ceratohyals) driven by elastic recoil of the pre-loaded, post-cranial tendons. (D) In seahorses (no data on snipefish), the hyoid can freely rotate as a result of the initially loose and folded nature of the mandibulohyoid ligament (see A). This ligament starts building up tension, causing the onset of mouth opening, and eventually abduction of the suspensoria. This results in snout widening, thereby generating suction. A–D are modified after Van Wassenbergh et al. (2013). E–G are based on Longo et al. (2018).

Pivot feeders evolved latch-mediated spring actuation (LaMSA) to power suction feeding indirectly with their body muscles (epaxials and hypaxials). The mechanisms in seahorses, pipefish (sygnathids) and snipefish are illustrated in Fig. 5. In seahorses, by preventing the hyoid from rotating its tip ventrally (Fig. 5A), the elevation of the neurocranium is also inhibited. This allows the epaxials and hypaxials to contract and store elastic energy in their well-developed tendons for a few tenths of a second (Van Wassenbergh et al., 2008, 2014). The latch is most likely the adducted suspensoria, which possesses a groove in which the hyoid fits and is prevented from rotating ventrally (Fig. 5B) (de Lussanet and Muller, 2007; Van Wassenbergh et al., 2013). A well-timed unlocking of the hyoid by slight abduction of the suspensoria by a trigger muscle causes a very fast dorsal pivoting of the head towards the prey, as well as a fast rotation of the small hyoid in the opposite direction (Fig. 5C,D). The relaxing adductor arcus palatini muscle and/or the contracting levator arcus palatini muscle are candidates for trigger muscles (de Lussanet and Muller, 2007; Van Wassenbergh et al., 2013). A different latch system and candidate trigger muscle have been identified for snipefish (Longo et al., 2018). In snipefish, the hyoid is initially positioned such that its tip would have to rotate dorsally if the neurocranium were to elevate (Fig. 5E). This keeps hyoid movement blocked and indirectly also inhibits elevation of the neurocranium (Fig. 5F). A trigger muscle, presumably the most ventral parts of the hypaxial muscles, may pull the hyoid tip ventrally to achieve unlocking (Longo et al., 2018) (Fig. 5G). The evolutionary origin of pivot feeding, and how differences in LaMSA methods evolved between syngnathiform fish (Fig. 5) remains partly unresolved.

The mouth cavity expansion system in seahorses (Fig. 5) is notably modified compared with that of a generalized teleost (Fig. 2). Unlike in other fish, the miniaturized hyoid causes negligible ventral expansion of the mouth cavity as it rotates ventrally (Roos et al., 2009; Van Wassenbergh et al., 2013). The opercula adduct during the initial stages of head rotation, and snout widening through suspensorium abduction follows relatively late in the feeding event (Roos et al., 2009) (Fig. 5D). The linkage systems enable seahorses to postpone mouth cavity expansion compared with the timing of neurocranial elevation. This is assumed to allow suction generation predominantly at times when the mouth is at a suitably close distance to the prey (Van Wassenbergh et al., 2013). Models showed that the same type of force transmission occurs at the level of the hyoid and suspensorium of seahorses (Van Wassenbergh et al., 2013), as in other teleosts (Aerts, 1991; Fig. 3A), but changes in the constructional parameters (Fig. 3B) and ligament and tendon slack (Figs 3 and 5) allow it to be tuned to the demands of pivot feeding.

Despite the need for powerful mouth expansion, power amplification does not seem to be common in suction-feeding fishes (Avidan et al., 2023). While it has been hypothesized in a few non-Sygnathiform species, it has not been conclusively demonstrated (Aerts et al., 1987; Grobecker, 1983; Grobecker and Pietsch, 1979; Ramsay and Wilga, 2017). Possibly phylogenetic or developmental limitations make it unlikely for the elements of elastic energy storage mechanisms (including a latch, energy storage structures such as tendons, necessary motor control) to evolve. Alternatively, suction feeding in many species may require a substantial magnitude of energy, so power amplification mechanisms that only increase the rate of energy (i.e. power) are insufficient.

### Muscle power: outlook and summary

Many open questions remain about how muscles power suction feeding and how muscle physiology interacts with skeletal kinematics and fluid dynamics. Directly measuring muscle power outputs during feeding is difficult. Mass-specific power has been estimated for relatively few species from muscle physiology (Carroll and Wainwright, 2006; Coughlin and Carroll, 2006) or from suction expansion power calculations (Avidan et al., 2023; Camp et al., 2015, 2018; Li et al., 2022; Van Wassenbergh et al., 2008). This has made it challenging to compare muscle function and suction power across species; however, existing data suggest intriguing variation (Avidan et al., 2023; Camp and Brainerd, 2022). Bluegill sunfish (*Lepomis macrochirus*) and royal knifefish (*Chitala blanci*) appear to be operating at or near their maximum power output during suction feeding (Camp et al., 2018; Jimenez and Brainerd, 2021; Li et al., 2022), while largemouth bass and channel catfish (*Ictalurus punctatus*) seem to be using only some of their potential power during feeding (Camp et al., 2015, 2020; Jimenez and Brainerd, 2020; Jimenez et al., 2023). Do these differences reflect trade-offs between swimming and feeding functions of the body muscles (Jimenez et al., 2021)? We hope future studies can integrate knowledge from feeding and swimming studies to better understand the structure, function and evolution of these muscles.

In summary, mouth expansion and suction flows are powered by active shortening of large regions of the body muscles (Camp and Brainerd, 2022). To meet these power demands (Avidan et al., 2023), fish body muscles must navigate force–velocity (Jimenez et al., 2021) and force–length trade-offs (Fig. 4). Here, the cranial muscles may play a crucial role, making lower-power motions that adjust kinematics, body muscle loading and ultimately flow patterns. The power amplification of pivot feeders demonstrates the tight integration of skeletal motion, muscle length and speed, and suction power and flows (Van Wassenbergh et al., 2013; Longo et al., 2018; Avidan et al., 2023). While amplification of body muscle power appears to be rare in suction-feeding fish, it provides a valuable system to explore the evolutionary morphology and biomechanics of powerful suction expansion.

## The interaction of musculoskeletal systems and fluid forces

**Fig. 6.**
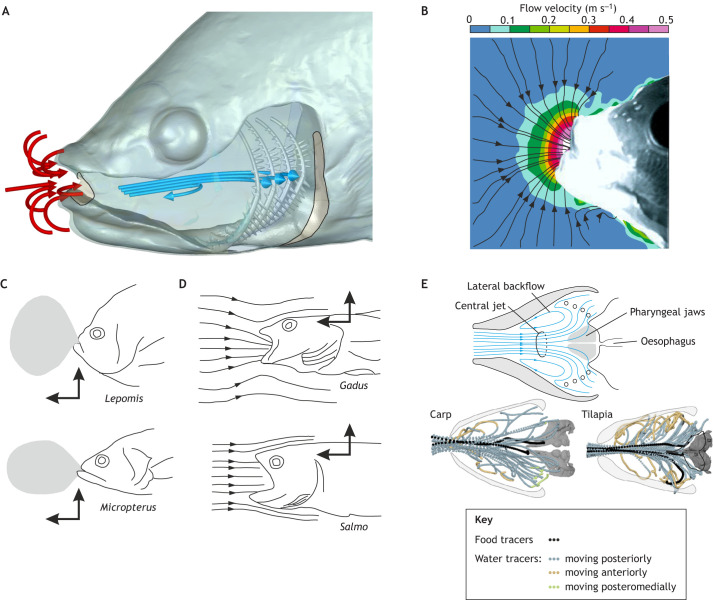
**Flow patterns during suction feeding.** (A) Illustration of typical extra-oral flows (red arrows) and intra-oral flow streamlines (blue arrows) relative to the fish's head during suction feeding. (B) Extra-oral flows that converge in three dimensions into the mouth opening are consistently observed in particle image velocimetry (PIV) studies (after Day et al., 2005). (C) The ingested volume of water in an earth-bound perspective during suction depends on ram speed [low in sunfish (*Lepomis*), higher in largemouth bass (*Micropterus*); redrawn from Higham et al., 2006]. (D) Flow streamlines, which capture a snapshot of instantaneous flow directions, from the fish's perspective become more parallel with increasing ram speed [low in cod (*Gadus*), higher in trout (*Salmo*); redrawn from Muller and Osse, 1984]. (E) Intra-oral flows have been quantified experimentally in carp (*Cyprinus*) and tilapia (*Oreochromis*), which showed a pathline pattern including a central jet and recirculation near the lateral sides (adapted from Provini et al., 2022; https://creativecommons.org/licenses/by/4.0/).

The short, powerful expansion of the head accelerates water and food towards, into and across the mouth cavity (Fig. 6A). These fluid flows and forces link suction-feeding kinematics to prey-capture performance (e.g. Day et al., 2005; Jacobs and Holzman, 2018; Muller and Osse, 1984; Muller et al., 1982; Ortega-Jimenez and Sanford, 2020; van Leeuwen, 1984; Yaniv et al., 2014). Successful suction feeding requires, first, that water carrying the food item is kept flowing from outside the fish, through the mouth and to the back of the mouth cavity. Second, the water must exit the mouth while the food is kept for processing or swallowing (Sanderson, 2024). Here, we summarize the suction-feeding flow patterns inside and outside the mouth and explore their relationship to skeletal kinematics and muscle power.

### Flows outside the mouth

A number of experimental studies have focused on the flow patterns in front of the mouth. Flow patterns in front of the mouth show a steep spatial gradient, only causing flow acceleration at a close distance from the mouth (Fig. 6B). A reason for this is that water is drawn from all directions towards an approximately circular opening of the mouth. When examining flows from an earth-bound perspective, studies using particle image velocimetry (PIV; see Glossary) discovered that flow patterns were stereotypical across fish species (Jacobs and Holzman, 2018), after being normalized to mouth size. Absolute suction flow speeds, however, vary significantly from below 0.1 m s^−1^ in small and weak suction feeders (Avidan et al., 2023) to over 2 m s^−1^ in powerful species (Ortega-Jimenez and Sanford, 2020). Several aspects of the hydrodynamics of suction in front of the mouth will depend on the ram speed (forward translation; Fig. 1C) (Muller and Van Leeuwen, 1985; van Leeuwen, 1984). The initial shape of the water that eventually becomes ingested by suction will be narrower and more stretched out with increasing ram speed (Higham et al., 2006; van Leeuwen, 1984; Fig. 6C). Along the same line, from the perspective of moving with the fish, ram speed will cause the streamlines (see Glossary) to become more parallel to the direction of movement (van Leeuwen, 1984; Weihs, 1980; Fig. 6D). Such differences in flow patterns for different sucking techniques have important implications for predator–prey interactions, such as affecting flow sensing and optimal escape direction in mobile prey (van Leeuwen and Muller, 1984; Stewart and McHenry, 2010; Gemmell et al., 2013; Stewart et al., 2014; Peterson and McHenry, 2022). Additional studies linking the characteristics of suction flows with prey-capture performance (e.g. Kane and Higham, 2014; Sommerfeld and Holzman, 2019) are important for enhancing our understanding of the ecological role of suction characteristics (Kane et al., 2019).

### Flows inside the mouth

Compared with flow patterns outside the mouth, less is understood about flow patterns within the oral cavity. Logically, these flows also stem from the mechanical power generated by the feeding muscles and therefore can vary significantly between fish. After food enters the mouth, it must reach the oesophagus at the back of the mouth cavity (Fig. 6E). During this time, water is also flowing through the branchial basket (see Glossary) and out of the mouth cavity. The gill rakers or pharyngeal jaws aid in retaining the food while water is expelled. Gill-arch structures, including the gill rakers, can also help retain food as water exits by acting as a sieve (Hoogenboezem et al., 1991) or a cross-flow filter (Divi et al., 2018; Sanderson et al., 2001; Witkop et al., 2023). Although some studies managed to visualize food paths (Provini et al., 2022; Sibbing et al., 1986; Weller et al., 2020), how fish transport food from the gill arches to the oesophageal sphincter remains unclear. A current review outlines unresolved questions concerning the role of the gill arches and gill rakers in the retention of small food particles (Sanderson, 2024).

A recent study found that intra-oral flow patterns are more complex than originally thought (Provini et al., 2022). Using X-ray tracing of neutrally buoyant particles, it was demonstrated that a simple, anterior-to-posterior flow filling the expanding mouth cavity does not occur in carp (*Cyprinus carpio*) or tilapia (*Oreochromis niloticus*). Instead, a considerable amount of backflow was noted near the lateral sides of the mouth cavity (Fig. 6E). This coincided with a centralized stream of water (a ‘central jet’) that extends relatively deep into the oral cavity, especially in tilapia. This flow pattern may help carry food more directly towards the pharyngeal jaw region (Provini et al., 2022). The generality of this pattern beyond medium-sized omnivorous fish, such as carp and tilapia, however, remains unknown.

These fluid flow patterns shape – and are shaped by – the morphology and motion of the feeding apparatus. Mouth expansion will be resisted by drag and added mass forces of the surrounding fluid and inertia of the cranial tissues, although these require a relatively small proportion of suction power (Aerts et al., 1987; Muller et al., 1982; Van Wassenbergh et al., 2015). Most of the power is required to accelerate fluid into the mouth with high rates of volume change and large changes in pressure relative to the environment. The relative contribution of rate of volume increase and change in pressure varies across species (Camp and Brainerd, 2022; Van Wassenbergh et al., 2006) and may reflect interactions between mouth shape, motion and fluid flows. After water momentum has been built up, flow into the mouth cavity will aid expansion (Osse and Muller, 1980). Forward movement (‘ram’) will reduce the required pressure change (see ‘translation pressure’ in Muller et al., 1982) for a given expansion, as less acceleration of the engulfed water is needed (Fig. 1B,C). Hence, ram facilitates expansion, which has implications for power demands. As mouth cavity expansion generally occurs in an anterior-to-posterior wave, this affects water flow and local power requirements (Muller et al., 1982). Intraoral flows will impact skeletal kinematics and loading late in mouth expansion, and cranial motions will shape the intraoral flows that transport food through the mouth and oesophagus (Weller et al., 2020). As we better understand how the musculoskeletal system interacts with fluid flows in and around the mouth, we will be able to more strongly link muscle and skeletal mechanics to suction-feeding performance.

## Conclusions

Our current knowledge provides a strong foundation in how fish meet the mechanical challenges of suction feeding. We propose that the biomechanics of suction feeding can be further understood by research on three key aspects. First, how the skeletal levers and linkages of the skull transform muscle shortening into 3D mouth cavity expansion. Second, how fish manage to use large portions of the body muscles to meet the power demands of suction feeding on elusive prey. Third, how mouth expansion shapes, and is shaped by, a continuous fluid flow that brings food from the outside, through the gape and to the back of the mouth cavity.

Discovering how the skeleton, muscles and fluid flows interact will provide new insights into the remarkable morphological, evolutionary and ecological diversity of fish suction feeding. The example of pivot feeding by syngnathiform fishes demonstrates well how successful suction depends on the interactions of the feeding skeleton, cranial and body muscles, and fluid flows (Fig. 5). Still, key aspects of the pivot-feeding mechanism, such as the triggering of their rapid strikes and the evolutionary transitions from ancestral, non-pivot feeders, remain hypothetical (Van Wassenbergh et al., 2013; Longo et al., 2018; Avidan et al., 2023). These LaMSA mechanisms are an extreme example of skeletal linkages impacting suction power. Other, more widespread innovations in the skeletal linkages of suction feeding – ­such as jaw protrusion (Wainwright et al., 2015) – may have less dramatic but still important roles in power production and transmission. We hope future studies will investigate this further.

This kind of integrative research is technically challenging. Yet, advances in experimental techniques such as X-ray reconstruction of moving morphology (XROMM) (Brainerd et al., 2010; Gatesy et al., 2010), flow visualization with PIV and computational modelling tools open new opportunities. We are particularly excited for future studies to test how skeletal linkages are ‘tuned’ to match muscle performance to fluid loading (Richards and Clemente, 2013), discover how body muscles navigate force–velocity trade-offs to deliver high power outputs, and determine how both fluid and musculoskeletal dynamics carry food through the mouth cavity. Future research that connects skeletal, muscular and fluid mechanics will be crucial for deepening our understanding of the morphology, physiology, evolution and ecology of suction feeding in fishes.
